# Study of the Interactions Between Bacteriophage phiIPLA-RODI and Four Chemical Disinfectants for the Elimination of *Staphylococcus aureus* Contamination

**DOI:** 10.3390/v10030103

**Published:** 2018-02-28

**Authors:** Seila Agún, Lucía Fernández, Eva González-Menéndez, Beatriz Martínez, Ana Rodríguez, Pilar García

**Affiliations:** Instituto de Productos Lácteos de Asturias (IPLA-CSIC), Paseo Río Linares s/n, 33300 Villaviciosa, Asturias, Spain; shei_s_s@hotmail.com (S.A.); eva.gm@ipla.csic.es (E.G.-M.); bmf1@ipla.csic.es (B.M.); anarguez@ipla.csic.es (A.R.); pgarcia@ipla.csic.es (P.G.)

**Keywords:** bacteriophages, disinfectants, food industry, *Staphylococcus aureus*

## Abstract

Bacteriophages are currently considered as a promising alternative to antibiotics and disinfectants. However, the use of phages in different clinical and industrial settings will involve their exposure to other disinfectants. As a result, the outcome of the phage treatment will depend on two aspects derived from such interactions. On the one hand, the susceptibility of the phage to disinfectants at the concentrations used for disinfection and at lower residual concentrations needs to be determined. Additionally, the existence of synergistic or antagonistic interactions between phages and disinfectants would also affect the potential success of phage biocontrol applications. Here, we tested these effects for the antistaphylococcal phage phiIPLA-RODI by using four different disinfectants: benzalkonium chloride, triclosan, chlorhexidine and hydrogen peroxide. Our results highlight the differences between disinfectants regarding their effect on phage survival and antimicrobial properties. For instance, our data suggests that, out of the four disinfectants used, benzalkonium chloride would be the most adequate to use in settings where phages are to be applied. Nonetheless, this preliminary analysis grants the need for further studies with a larger number of disinfectants for the development of a phiIPLA-RODI-based product.

## 1. Introduction

The use of bacteriophages as antimicrobials was proposed almost 100 years ago by Félix d’Herelle [1]. However, the introduction of antibiotics shortly afterwards temporarily halted the use of the so-called phage therapy in Western countries [2]. In the meantime, bacterial viruses have been isolated and collected in Eastern Europe, where phages have continued to be administered as antibacterials [2]. More recently, due to the relentless increase in bacterial resistance to conventional antimicrobials, the potential use of phages has been reconsidered [2]. As a result, numerous studies are currently investigating the efficacy of bacteriophage-based products to eliminate or reduce bacterial contamination in different settings. One possible application is the use of phage products for disinfection of surfaces in hospital or food industry settings [3,4,5]. Indeed, some phage-based products have already been developed and placed on the market. For instance, there are two commercial products against *Listeria monocytogenes* contamination, ListShield^TM^ and PhageGuard Listex, which are respectively manufactured by Intralytix Inc. (Baltimore, MD, USA) and Micreos BV (Wageningen, The Netherlands). Similarly, products containing bacteriophages against *Escherichia coli* O157:H7 and *Salmonella* sp. manufactured by Intralytix Inc. have been recently approved by the FDA.

During the application of phage-based surface disinfectants, bacteriophages will almost certainly encounter other antimicrobial compounds that are used as part of routine cleaning and disinfection procedures. With this in mind, analysis of these interactions is an important part of the development of phage-based products. Indeed, the results of such preliminary assessment may determine the selection of a given bacteriophage rather than others or provide information regarding the instructions for the application of the end product or even its composition. For instance, it is very important to determine the susceptibility of bacteriophages to disinfectants for two reasons. On the one hand, we do not want our “phage of choice” to be inactivated due to disinfection protocols performed on the treated surface. This would render our phage product essentially useless in the presence of disinfectants, an information that must be indicated on the product label. On the other hand, due to regulatory constraints, it may be of interest to determine disinfection procedures that can be used to inactivate the phage before using the treated surface. This is important, for example, in the food industry as the authorization approval of certain phage-based products may require that no active phages reach the consumer. In addition to phage susceptibility to disinfectants, another important issue is the potential occurrence of synergistic or antagonistic interactions between the virus and chemical disinfectants. In that sense, the presence of residual concentrations of disinfectants prior to phage application might affect the efficacy of the phage product even if they do not inactivate the viral particles.

To date, the effect of industrial disinfectants on bacteriophages has been studied mostly for viruses that infect lactic acid bacteria [6,7]. This is not surprising since phages have consistently represented an significant source of problems in dairy production [5,8]. Also, a study has evaluated the persistence of phages encoding the Shiga toxin after different disinfection treatments [9]. Overall, these studies seem to indicate that resistance to different disinfectants needs to be examined for each specific bacteriophage. However, there is no current information available regarding the ability of bacteriophages with antimicrobial potential to withstand exposure to biocides. Instead, a few studies have assessed the potential use of bacteriophages in combination with chemical antimicrobials for reducing bacterial contamination [10,11].

The elimination of the recalcitrant biofilms formed by pathogenic bacteria on biotic and abiotic surfaces is perhaps one of the most interesting settings for the use of bacteriophages as disinfectants [4,12]. These structures are resistant to most traditional antimicrobial strategies, making it necessary to design and implement new protocols [13]. In this regard, this study set out to examine the potential interactions between a phage infecting *Staphylococcus aureus* and four selected disinfectants that have distinct chemical structures and/or modes or action. vB_SauM_phiIPLA-RODI (phiIPLA-RODI) is a virulent myophage with known efficacy regarding the removal of biofilms formed by different *S. aureus* strains alone or in combination with other microorganisms [14,15]. Elimination of staphylococcal biofilms is very important within the context of both hospital and industrial settings, as they can represent a serious health threat [4]. In the food industry, for example, *S. aureus* can lead to infections as well as to intoxications due to the production of heat-stable enterotoxins. Therefore, the increased protection of *Staphylococcus* cells inside biofilms against disinfection strategies is a cause of concern. In this scenario, phiIPLA-RODI seems a good candidate for the development of phage-based products against biofilms formed by *S. aureus* on food industry surfaces. Nonetheless, one of the steps involved in developing such products is the assessment of the impact of disinfectants used in the food industry on the effectiveness of phiIPLA-RODI as an antimicrobial and antibiofilm agent. Our results provide preliminary data on this topic and hint that some widely used disinfectants should be avoided in order to ensure the efficacy of products based on phage phiIPLA-RODI.

## 2. Materials and Methods

### 2.1. Bacterial Strains and Phages Used and Growth Conditions

All experiments were performed using *S. aureus* IPLA1 [16] and bacteriophage phiIPLA-RODI [14]. Propagation and titration of the phage were performed as previously described [14]. *S. aureus* was routinely grown on TSB medium (Tryptic Soy Broth, Scharlau, Barcelona, Spain) at 37 °C in an orbital incubator or on TSA plates, containing TSB supplemented with 2% agar. The disinfectants used were hydrogen peroxide (VWR International S.A.S., Fontenay-sous-Bois, France), benzalkonium chloride (Sigma-Aldrich Chemical Co., St. Louis, MO, USA), chlorhexidine digluconate (Sigma-Aldrich Chemical Co., St. Louis, MO, USA) and triclosan (Sigma-Aldrich Chemical Co., St. Louis, MO, USA). All concentrated stocks of disinfectants were prepared in water except triclosan, which was dissolved in 70% ethanol at a concentration of 1 mg/mL. These disinfectants were chosen based on their different chemical structure and mode of action (Table 1) [17]. Moreover, these four compounds are widely used in the formulation of products used for cleaning and disinfection in industrial and/or hospital settings, as well as in products aimed for household cleaning and personal hygiene. As a result, their interaction with bacteriophages would be relevant for the development of phage-based antimicrobial products.

### 2.2. Phage Susceptibility to Different Disinfectants

To determine phage susceptibility to different disinfectants, a phage suspension was diluted to a final concentration of approximately 10^5^ PFU/mL in TSB and different final concentrations of chemical antimicrobial agents or no antimicrobial (control) were added. These suspensions were then incubated overnight at 37 °C. The following day, the surviving phage particles in each sample were determined by the double-layer assay. The survivor particles in the treated samples were compared to the control and used to calculate the percentage of survival. It must be noted that residual amounts of disinfectants in the titrated samples did not inhibit growth of the bacterial lawn.

### 2.3. Minimal Inhibitory Concentration (MIC) Determination and Checkerboard Assays

Determination of the minimal inhibitory concentration (MIC) values for the disinfectants and the minimum inhibitory multiplicity of infection (MOI) values for the phage was performed according to the broth microdilution technique following the CLSI guidelines [18,19] but using TSB as a growth medium. The MIC, and the minimum inhibitory MOI for the phage, was determined as the lowest concentration of the antimicrobial that inhibited visible bacterial growth after 24 h of incubation at 37 °C. All MIC assays were repeated 5 to 8 times.

To determine whether there was synergistic or antagonistic interaction between the phage and the disinfectants, the checkerboard method was used [20]. This technique is a modification of the broth microdilution method, in which the bacterium is exposed to two antimicrobials diluted in a two-dimensional fashion. In this case, cell suspensions of *S. aureus* IPLA1 were exposed to combinations of a chemical disinfectant and different concentrations of phage phiIPLA-RODI. Following incubation for 24 h at 37 °C, bacterial growth in the different wells was assessed and used to determine the potential existence of interactions. To do that, the fractional inhibitory concentration (FIC) for each antimicrobial was calculated as described previously [21]. These values were then used to determine the FIC index (FICi), which is calculated as the sum of the FICs for a given well. FICi values below 0.5 were considered to represent synergy and values above 4.0 were considered an indication of antagonism. Otherwise, it was determined that there was no interaction between the two antimicrobials.

### 2.4. BIMs

Bacteriophage-insenstive mutants (BIMs) were isolated as previously described [14]. Briefly, overnight cultures of strain *S. aureus* IPLA1 were grown in the presence of subinhibitory concentrations of clorhexidine at 37 °C with shaking. A control was grown in culture medium alone. 100 μL aliquots from these overnight cultures were taken (10^8^ CFU) and incubated with 100 μL of phage stock (10^9^ PFU) for 10 min at 37 °C. Then, this mixture was combined with 3 mL of soft agar and poured onto thin TSA plates. These plates were incubated overnight at 37 °C. The number of surviving colonies was counted the following day to calculate the frequency of BIMs by dividing this number by the initial number of bacteria inoculated on the plate.

### 2.5. Phage Adsorption Rate

To determine the phage adsorption rate, an overnight culture of *S. aureus* IPLA1 was diluted 1:100 into fresh TSB medium containing different concentrations of chlorhexidine (0, 0.25 and 0.5 μg/mL) and grown to an OD_600_ of 1. 900 μL aliquots from these cultures (~10^8^ CFU/mL) were then mixed with 100 μL of a phage stock containing 10^7^ PFU/mL (MOI = 0.1). A control sample was prepared by mixing 900 μL of sterile TSB with 100 μL of phage stock. All samples were incubated for 5 min at room temperature to allow for phage adsorption to occur. After incubation, samples were centrifuged for 3 min at 10,000× *g* at 4 °C and the supernatants were collected and titrated following the double layer technique to determine the number of non-adsorbed phages. The phage adsorption rate for each sample was calculated by using the following formula: phage adsorption rate = [(phage number in supernatant of control − phage number in supernatant sample)/(phage number in supernatant of control)] × 100.

### 2.6. Biofilm Formation and Treatment Assays

In order to perform the biofilm treatment assays, biofilms were first preformed. Thus, overnight cultures of *S. aureus* IPLA1 were diluted down in TSB supplemented with 0.25% glucose to obtain a cell suspension containing 10^6^ CFU/mL. 200 μL from this suspension was used to inoculate each well of a Nunclon Delta polystyrene 96-well microtitre plate (Thermo Scientific, NUNC, Madrid, Spain). Following incubation at 37 °C for 24 h, the planktonic phase was removed from the wells and the biofilms were washed twice with PBS. Then, TSB containing different combinations of disinfectant concentrations plus bacteriophage (10^9^ PFU/mL) were added to the wells. Treatment was performed for 24 h at 37 °C. After treament, the biofilm was washed again with PBS and subsequently stained with crystal violet as previously described [22]. Briefly, 200 μL of 0.1% crystal violet were added to each well and subsequently removed after incubation for 15 min at room temperature. Then, the excess dye was removed by washing twice with distilled water. Finally, 200 μL of 33% acetic acid was added to each well to solubilize the dye prior to quantification by measuring A_595_ in a Benchmark Plus microplate spectrophotometer (Bio-Rad Laboratories, Hercules, CA, USA). Biofilm removal was determined as the percentage of biomass found in samples treated with phage and/or disinfectant compared to the biomass of the untreated control (treated with medium alone) according to the equation [A_595_ (treated sample)/A_595_ (untreated control sample)] × 100.

### 2.7. Statistical Analysis

Data obtained from at least three independent biological replicates were analyzed with a two-tailed Student’s *t* test. *p*-Values < 0.05 were considered significant.

## 3. Results

### 3.1. Susceptibility of Phage phiIPLA-RODI to Different Disinfectants

#### 3.1.1. Susceptibility at Concentrations Used in Household and Industrial Products

The first objective of this study was to determine whether four different antimicrobial compounds led to the inactivation of the *S. aureus* phage phiIPLA-RODI. The compounds selected for this analysis were: benzalkonium chloride, chlorhexidine, hydrogen peroxide and triclosan, four disinfectant agents belonging to different chemical classes (Table 1). The concentrations tested are in the range of those present in commercial products aimed for household or industrial disinfection procedures. In most cases, overnight incubation with these disinfectants led to inactivation of the phage particles in the suspension to undetectable levels (Table 2). One exception was 0.02% chlorhexidine, a concentration used in catheter maintenance solutions, in which 96.33% of the phage particles were inactivated. Likewise incubation in triclosan at 0.03% or 0.003% led to inactivation of 97.37% and 78% of the phage particles, respectively.

#### 3.1.2. Susceptibility at Disinfectant Concentrations Near the MIC of the Host Bacterium

Before studying the susceptibility of phiIPLA-RODI to low concentrations of the disinfectants, it was necessary to assess the susceptibility of *S. aureus* IPLA1 to the four compounds. The determined MIC values for benzalkonium chloride, triclosan, chlorhexidine and hydrogen peroxide were 1 μg/mL, 0.25 μg/mL, 2 μg/mL and 0.6 mM, respectively.

**Figure 1:**
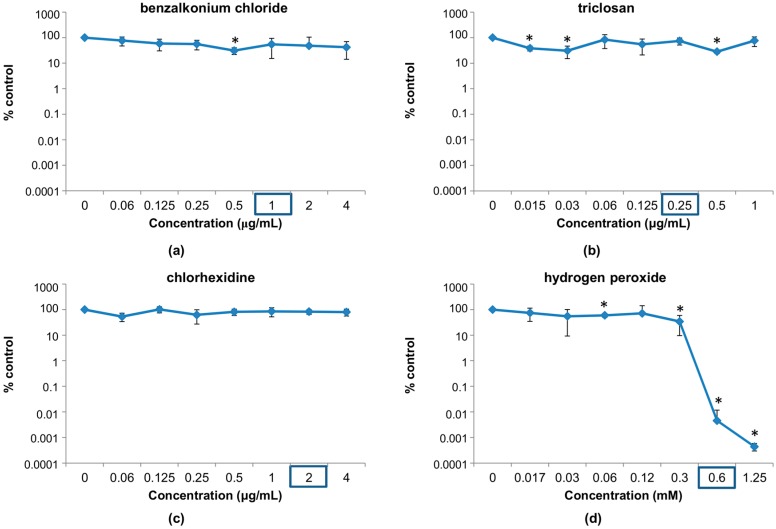
Percentage of survival of phiIPLA-RODI phage particles after overnight treatment with different concentrations of disinfectants: (**a**) benzalkonium chloride; (**b**) triclosan; (**c**) chlorhexidine; (**d**) hydrogen peroxide compared to an untreated control without disinfectant. The minimal inhibitory concentrations (MIC) for *S. aureus* IPLA1 for each disinfectant is marked with a blue rectangle. * *p*-Value < 0.05.

Taking these values into account, we tested the viability of the phage following incubation with antimicrobial concentrations near and below their respective MICs. In this range, the results were quite different from those observed in the previous experiment (Figure 1). Indeed, no major decrease in phage survival (more than one logarithmic unit) was observed for most of the concentrations used in the test. A notable exception to this is hydrogen peroxide, which can significantly inactivate the phage at doses near the MIC for *S. aureus* IPLA1 (Figure 1d). In fact, a decrease of more than 4 or 5 logarithmic units was observed at 0.6 and 1.25 mM, respectively. For chlorhexidine, however, none of the concentrations used here led to a significant reduction in phage counts (Figure 1c). In the case of benzalkonium chloride, only a concentration of 0.5 μg/mL led to a minor decrease in phage survival (Figure 1a). Finally, triclosan led to small reductions (<90%) in phage survival at different concentrations, more specifically at 0.015, 0.03 and 0.5 μg/mL (Figure 1b).

### 3.2. Interactions between Phage phiIPLA-RODI and Disinfectants

**Figure 2:**
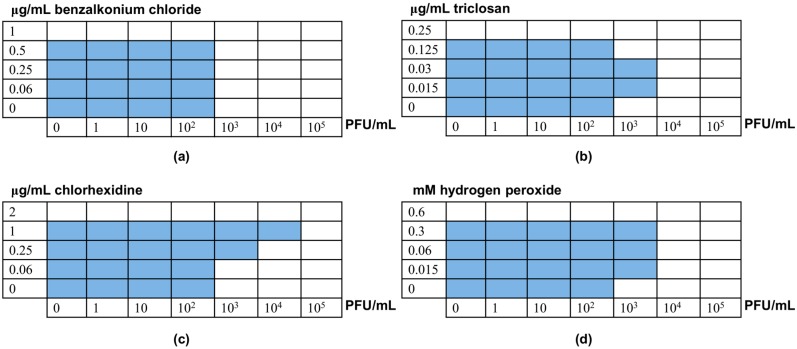
Results of the checkerboard assays to test the interactions between phage phiIPLA-RODI and the following disinfectants: (**a**) benzalkonium chloride; (**b**) triclosan; (**c**) chlorhexidine; (**d**) hydrogen peroxide. Wells with bacterial growth after 24 h of incubation at 37 °C are marked in blue.

Besides affecting phage viability, the presence of chemical disinfectants may alter the antimicrobial effect of the bacteriophage either positively (synergy) or negatively (antagonism). This phenomenon would have an impact on the efficacy of phage-based products. Therefore, it is important to establish the existence of such interactions between a specific bacteriophage and different disinfectants that it may encounter during its application. With this in mind, we tested if residual concentrations of the four disinfectants used in this study affected the outcome of bacteriophage-bacteria interactions. Our results varied depending on the specific disinfectant (Figure 2). First of all, it must be noted that the presence of the phage did not affect susceptibility of *S. aureus* IPLA1 to any of the four disinfectants tested. In contrast, some of the disinfectants did alter the ability of the phage to inhibit bacterial growth. The most obvious changes were observed in the presence of chlorhexidine, an antimicrobial that did not have an impact on phage survival after overnight incubation (Figure 1c). In spite of this, subinhibitory doses of chlorhexidine led to an increase in the MIC to phiIPLA-RODI, thereby indicating the existence of antagonism between the virus and the disinfectant (Figure 2c). Indeed, ½ × MIC increased resistance to the phage by 100-fold. In the case of triclosan and hydrogen peroxide, some subinhibitory concentrations increased the MIC to phage phiIPLA-RODI by 10-fold (Figure 2b,c). However, it must be noted that, unlike chlorhexidine, these two compounds did affect phage survival (Figure 1b,c). Consequently, the results observed in the checkerboard assays may reflect this loss of viability after incubation of the phage with the disinfectants. Finally, the quaternary ammonium compound benzalkonium chloride did not have an impact on bacterial susceptibility to the virus (Figure 2a). The FIC index calculated for benzalkonium chloride and the phage was 2, which indicates no interactions between the two antimicrobials. In contrast the FIC index for the other disinfectants and the phage was 11, indicative of antagonism.

Since the result observed for chlorhexidine could not be explained by reduced survival of the phage due to the disinfectant, we tried to identify the mechanism behind the antagonistic effect between the two antimicrobials. One of the possible explanations would be that exposure to subinhibitory concentrations of chlorhexidine increases the frequency of BIMs in the bacterial population, leading to greater resistance to the phage. However, the average frequency of BIMs did not vary significantly in populations exposed to 0.25 μg/mL, 0.5 μg/mL or 1 μg/mL of chlorhexidine compared to the control sample without antimicrobial (Table 3). Another potential explanation for this phenomenon may be that exposure to subinhibitory chlorhexidine affects the bacterial cell in such a way that phage adsorption is limited. We tested this hypothesis by growing *S. aureus* IPLA1 in two subinhibitory concentrations of chlorhexidine that did not significantly affect bacterial growth or decrease phage viability. The results did not show any significant change in the phage adsorption rate in the samples grown with the disinfectant (Table 3).

These results highlight that the interactions between bacteriophages and chemical antimicrobials may vary and do not always correlate with the susceptibility of the phage particles to a specific compound. As evidenced by the results obtained for chlorhexidine, bacterial cells may exhibit differences in phage susceptibility when exposed to subinhibitory concentrations of certain disinfectants. Further work is still necessary, however, to determine the specific mechanisms involved in this phenomenon.

### 3.3. Elimination of S. aureus Biofilms by Combinations between Phage phiIPLA-RODI and Disinfectants

**Figure 3:**
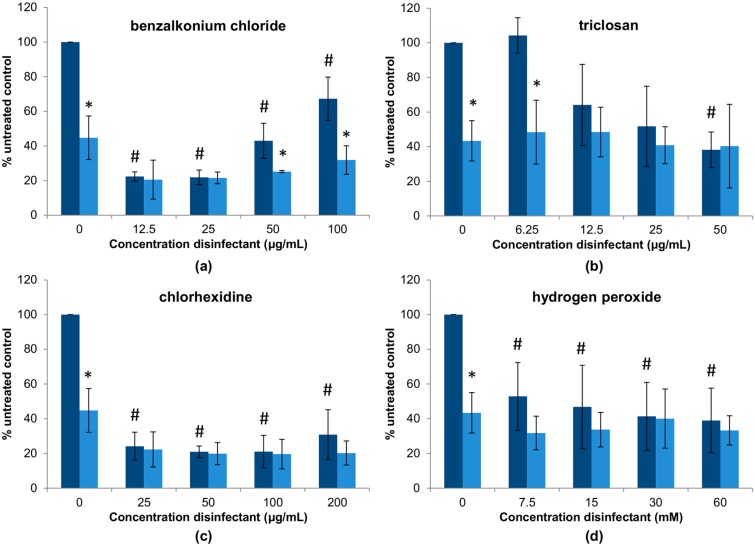
Representation of the data obtained in the biofilm removal assays to test the interactions between a high dose of phage phiIPLA-RODI (10^9^ PFU/mL) and different concentrations of the following disinfectants: (**a**) benzalkonium chloride; (**b**) triclosan; (**c**) chlorhexidine; (**d**) hydrogen peroxide. Dark blue and light blue bars represent samples without or with phage treatment. Values obtained without phage treatment were compared to those treated with phiIPLA-RODI and *p*-values < 0.05 were considered significant (*). Additionally, values obtained with disinfectant were compared to the untreated control to determine the antibiofilm effects of the four antimicrobials and *p*-values < 0.05 were considered significant (#).

In the previous experiment, we explored how different combinations of a bacteriophage and disinfectants affect antimicrobial activity of the phage on planktonic cells in a standard broth microdilution method. However, these interactions may be different when it comes to biofilm elimination. To examine this possibility, preformed 24-h biofilms of *S. aureus* IPLA1 were treated with different combinations of phiIPLA-RODI and the four disinfectants (Figure 3). An initial observation of the results reveals that, out of the four disinfectants, triclosan and hydrogen peroxide seem to be the least effective for biofilm removal (Figure 3b,d), followed by benzalkonium chloride (Figure 3a) and chlorhexidine (Figure 3c), with the latter showing very good antibiofilm activity at all concentrations tested. Of note, treatment with higher doses of benzalkonium chloride was less effective for biofilm elimination than lower concentrations. Indeed concentrations of 12.5 μg/mL and 25 μg/mL led to a reduction in attached biomass of approximately 80%, whereas concentrations of 50 μg/mL and 100 μg/mL decreased biofilm by ~40–60% (Figure 3a). Perhaps, despite the bactericidal activity of the disinfectant, these concentrations promote biofilm formation; thereby resulting in a lesser reduction of the attached biomass. Triclosan was only effective for biofilm removal at the highest concentration tested (50 μg/mL), with an elimination of approximately 60% of the biomass (Figure 3b). Lower concentrations showed no reduction at all (6.25 μg/mL) or only slight reductions of up to 40–50%, with high variability between experiments, at 12.5 μg/mL and 25 μg/mL. Biofilm removal by chlorhexidine was about 80% for all concentrations analyzed (Figure 3c), while the average biomass reduction with hydrogen peroxide was approximately 50–60% at concentrations between 7.5 and 60 mM (Figure 3d).

Treatment with the phage alone, without disinfectant, led to a decrease in attached biomass of approximately 43% compared to the untreated control. In most cases, adding the virus did not significantly improve treatment with the chemical disinfectants. However, there are some exceptions to this. For instance, combination of the phage with benzalkonium chloride significantly improved treatment at higher concentrations (50 μg/mL and 100 μg/mL) of the disinfectant (Figure 3a). Therefore, addition of phiIPLA-RODI could counteract the reduced efficacy of benzalkonium chloride at these doses compared to lower concentrations. Regarding triclosan, the lowest concentration tested (6.25 μg/mL) did not exert a significant ability to eliminate biofilms (Figure 3b). In contrast, that same concentration together with phiIPLA-RODI did, with a difference akin to that obtained with the bacteriophage alone. This suggested that the virus was not significantly affected by the chemical during biofilm treatment even though the phage was inactivated by the same range of triclosan concentrations when incubated in a suspension (Table 2). It is thus possible that the viral particles are protected from the antimicrobial agent by the extracellular matrix or inside host cells. Alternatively, phage infection and propagation might occur at a faster rate than phage inactivation by triclosan, thereby balancing out the virucidal effect of the disinfectant. In the case of chlorhexidine and hydrogen peroxide, the presence of the phage did not alter the efficacy of the disinfectants (Figure 3c,d).

Taken together, all these assays show that, in order to get a clear picture of the interactions between bacteriophages and disinfectants, it is important to examine such interactions from different perspectives. Thus, in addition to assessing phage susceptibility to a given compound, it is necessary to determine how the virus and the chemical agent will mutually affect each other in the presence of bacterial cells. Moreover, the interplay between disinfectants, phages and bacteria has to be analyzed for planktonic and biofilm cells given the notable differences between these two states.

## 4. Discussion

Bacteriophages have great potential as antimicrobial agents. Their specificity, together with their safety regarding human health and the environment, makes them a very interesting option to complement routine cleaning and disinfection procedures. However, the use of bacterial viruses in industrial and medical settings will necessarily involve their interaction with chemical disinfectants. Such interactions may affect the efficacy of bacteriophages as antimicrobials and, as a result, should be examined prior to the development of phage-based products. With this in mind, this study aimed to obtain some preliminary information regarding the effect of four common chemical disinfectants on a promising antistaphylococcal phage. Previous studies had shown that virulent phage phiIPLA-RODI is able to infect and lyse a wide range of *S. aureus* strains, not only during planktonic growth but also when forming biofilms, even with other species [14,15].

One of the aspects explored here is the susceptibility of phiIPLA-RODI to four disinfectants with different chemical structures. To date, most information regarding phage susceptibility to disinfectants has been gathered for phages infecting lactic acid bacteria (LAB), which are a problem in the dairy industry [6,7]. Conversely, information on the efficacy of commonly used biocides against phages that inactivate bacterial pathogens is scarce. Our results showed that exposure of phiIPLA-RODI to concentrations in the range of industrial or household biocidal products led to significant reductions in the numbers of surviving viral particles. However, there were notable differences between antimicrobial agents regarding their antiviral properties. Thus, benzalkonium chloride and hydrogen peroxide eliminated the virus to undetectable levels at all concentrations tested, whereas some concentrations of chlorhexidine and triclosan did not. In contrast, when the phage was challenged with lower, residual concentrations of the disinfectants, closer to the MIC of the bacterium, only hydrogen peroxide led to a major reduction (>99%) in the number of viral particles. Interestingly, QACs and oxidizing agents were the most effective against several LAB phages [6]. In another study, the most effective biocide against lactococcal phages was benzalkonium chloride followed by hydrogen peroxide, whereas other biocides were only active against some phages [7]. Despite the apparent trends of certain disinfectants to be more effective for the inactivation of bacteriophages, it does seem evident that this is a property that needs to be examined for each phage with antimicrobial potential. In view of our results, it seems that phage-based products should not be applied together with chemical disinfectants at routine working concentrations, although lower doses of the biocides can be tolerated by the phage while retaining considerable antibacterial activity. It can also be pointed out that biocidal treatment with benzalkonium chloride or hydrogen peroxide could be used to inactivate the phage in order to comply with the strict current regulations regarding phage applications. As a result of this, no viable phage particles would reach, for example, food products that subsequently come into contact with the phage-treated surfaces.

Given the fact that low concentrations of most antimicrobial agents tested are rather innocuous for the phage, it seemed interesting to explore the existence of potential synergistic interactions against planktonic cells of *S. aureus*. However, the data obtained in checkerboard assays showed no such synergy. Indeed, some antimicrobials demonstrated an additive effect with the phage, while others exhibited a clear antagonism. In some cases, the observed antagonism may be a result of partial phage inactivation by the biocide at the concentrations tested, as is the case of hydrogen peroxide and triclosan. Surprisingly, the most clear antagonistic interaction occurred between phiIPLA-RODI and chlorhexidine, despite the fact that low concentrations of this disinfectant did not alter phage particle viability. This result suggests the existence of a secondary effect of chlorhexidine that interferes with the development of phage infection. Our results suggest that this phenomenon is not due to an increased presence of phage resistant mutants or to a decrease in the adsorption rate when bacteria have been exposed to chlorhexidine. However, regardless of the mechanism responsible for the antagonism observed, it seems that chlorhexidine, even at very low concentrations, should not be used in environments where phage treatment is to be performed.

Given the relevance of biofilms formed on different biotic and abiotic surfaces, the interactions between the bacteriophage and the disinfectants regarding biofilm removal were also assessed. In general, our results confirm the potential of phiIPLA-RODI for eliminating biofilms formed by *S. aureus* observed in previous studies [14,15]. Moreover, the viral particles seemed to withstand higher concentrations of disinfectants when infecting bacterial biofilms compared to liquid cultures. Thus, the biofilm architecture appears to protect not only bacterial cells, but also their predator phages, from antimicrobial agents. In most of the combinations used, there was no significant change from the presence of the two antimicrobials, phage and chemical agent. However, the bacteriophage improved removal when combined with some concentrations of triclosan or benzalkonium chloride.

Overall, the results obtained in this study have highlighted the importance of evaluating the impact of cleaning and disinfecting agents on the efficacy of a phage-based antimicrobial product. Moreover, the data presented here provide hints regarding the design and label indications for the production of a new antimicrobial based on phiIPLA-RODI against *S. aureus*. Nonetheless, the diversity of the responses depending on the specific disinfectant and its concentration also indicates that this preliminary study deserves to be followed by further analysis including more disinfectants.

## Figures and Tables

**Table 1:** Disinfectants chosen for this study.

Disinfectant	Chemical Class	Mode of Action
**Benzalkonium chloride** 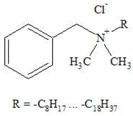	Quaternarium ammonium compound (QAC)	Membrane damage
**Chlorhexidine** 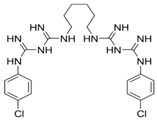	Biguanide	Membrane damage
**Hydrogen peroxide** 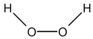	Oxidizing agent	Oxidative damage
**Triclosan** 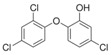	Phenolic compound	Membrane disruption, FabI inhibition

**Table 2:** Survival of phage phiIPLA-RODI in the presence of disinfectants.

Disinfectant	Concentration	% Survival Compared to Untreated Control
Benzalkonium chloride	0.002% (20 μg/mL)	<0.02
	0.02% (200 μg/mL)	<0.02
	5% (50 mg/mL)	<0.02
Chlorhexidine	0.02% (200 μg/mL)	3.67 ± 2.07
	0.2% (2 mg/mL)	<0.02
Hydrogen peroxide	0.3% (80 mM)	<0.02
	3% (0.8 M)	<0.02
Triclosan	0.003% (30 μg/mL)	22.00 ± 7.21
	0.03% (300 μg/mL)	2.63 ± 0.94

**Table 3:** Effect of chlorhexidine on frequency of BIMs and phage adsorption rate of phiIPLA-RODI.

Chlorhexidine Concentration	BIM Frequency	Phage Adsorption Rate
0 μg/mL	3.79 × 10^−7^	87.22 ± 3.16
0.25 μg/mL	3.55 × 10^−7^	84.08 ± 14.17
0.5 μg/mL	3.09 × 10^−7^	89.00 ± 5.36
1 μg/mL	2.34 × 10^−7^	Not determined
